# 

**Published:** 1869-01-15

**Authors:** 


### CHICAGO MEDICAL JOURNAL.

J. ADAMS ALLEN, M.D., LL.D., Editor. WALTER HAY, A.M., M.D., Associate Editor.

All letters for THE JOURNAL should be addressed to DR. J. ADAMS ALLEN, No. 71 Dearborn Street, Chicago, Ill.

TERMS—$3 PER ANNUM, IN ADVANCE.

$4 if not paid before 1st of July, in each year. Single Copies, 25 Cents.

Wythe's Physicians' Pocket Dose and Symptom Book.......... 64

Cleaveland's Pronouncing Medical Lexicon.......... 64

RUSH MEDICAL COLLEGE—SPRING ANNOUNCEMENT.......... 64

CHICAGO, Jan. 25, 1869.

The Association of the Alumni of Rush Medical College will hold its first annual meeting Commencement day, February 3, 1869, at 2 o'clock P. M., in the upper lecture room of the College.

All graduates, and all who have received the honorary or *ad eundem* degree in the College, are requested to be present.

Such as have never signed the constitution of the Association, are requested to call at once on the Secretary, in the College building, that he may procure their address.

A Social Festival will be had in the evening at the house of Dr. W. C. Hunt.

W. C. HUNT, Secretary.

A TREATISE ON THE PRINCIPLES OF MEDICINE AND PATHOLOGY, DISEASES OF WOMEN AND CHILDREN, AND MEDICAL SURGERY. By W. PAINE, M. D. Philadelphia: *University Publishing Society.*

THE PHILADELPHIA SYSTEM OF OBSTETRICS. IN TWELVE PARTS. FULLY ILLUSTRATED. Designed for a Text-Book for Students. and as a Reference for the Practitioner. By JOS. S. LONGSHORE, M. D., Professor of Obstetrics, etc. Philadelphia: *University Publication Society.*

CLINICAL LECTURES ON DISEASES OF THE URINARY ORGANS, DELIVERED AT UNIVERSITY COLLEGE HOSPITAL. By SIR HENRY THOMPSON, Surgeon Extraordinary to H. M. the King of Belgians; Professor of Clinical Surgery, and Surgeon to University College Hospital. With Illustrations. Philadelphia: *Henry C. Lea,* 1866. Pp. 204. Chicago: *S. C. Griggs & Co.*

The subjects very pleasantly and lucidly discussed are: Diagnosis, Urethral Structures, Hypertrophy of the Prostate, Retention of Urine, Extravasation of Urine and Urinary Fistulæ, Stone in the Bladder, Lithotrity, Lithotomy, Cystitis and Prostatitis, Diseases of the Bladder, Paralysis, Atony, Juvenile Incontinence, Tumors, Hæmaturia and Renal Calculus.

ON CHRONIC BRONCHITIS—ESPECIALLY AS CONNECTED WITH GOUT, ELPHYSEMA, AND DISEASES OF THE HEART. Being Clinical Lectures delivered at the Middlesex Hospital, by E. HEADLAM GREENHOW, M.D, Fellow of the Royal College of Physicians, Consulting Physician of the Western General Dispensary, &c., &c. One volume. Octavo. Price, $2.25.—Chicago: *W. B. Keen & Co.*

MACKENZIE ON THE USE OF THE LARYNGOSCOPE IN DISEASES OF THE THROAT. Second Edition, with additions, and an Essay on Hoarseness, Loss of Voice, and Stridulous Breathing in relation to Nervo-Muscular affections of the Larynx. Edited by J. Solis Cohen, M.D., and Illustrated by two lithograpic plates, and fifty-one engravings on wood. Octavo. Price, $3.00. Chicago: *W. B. Keen & Co.*

WYTHE'S PHYSICIANS' POCKET DOSE AND SYMPTOM BOOK.—Eighth edition, 32mo., cloth. Price, $1.00.
do do leather, with tucks and pockets, $1.25. Chicago: *W. B. Keen & Co.*

CLEAVELAND'S PRONOUNCING MEDICAL LEXICON. A new and improved edition (the eleventh). Price, $1.25. Chicago: *W. B. Keen & Co.*

### *Rush Medical College—Spring Announcement.*

The Session will commence Wednesday, March 3d, 1869, and continue to the 1st of July.

The Course will consist of daily lectures and recitation at the College, and Clinical observations in the Hospitals, Infirmaries, and Dispensaries of the City.

Prof. BLANEY will give instructions in Practical Chemistry, affording access for that purpose to the large and well-appointed Laboratory of the College.

Prof. POWELL will teach Surgery.

Prof. GUNN will conduct his Surgical *Clinique* at the College.

Prof. ROSS will conduct the Medical *Clinique* and instruct in Physical Diagnosis and Diseases of the Chest.

The County Hospital will afford two Medical and Surgical *Cliniques* each week.

The Marine Hospital will be open to students of the Course.

The Charitable Eye and Ear Infirmary is located near the College and will be open every day of the week for Clinical teaching, by E. L. Holmes, M. D., Lecturer on Disease of the Eye and Ear.

The College Dispensary will be open every day, affording to students an excellent opportunity for perfecting themselves in the art of diagnosis.

The Dissecting Room will be kept open during the entire session, and will be abundantly supplied with material for the study of Practical Anatomy.

Special instruction in Practical Chemistry will be given by Prof. Blaney, to such as desire.

Graduates of the College will be admitted to the Spring Session on the matriculation ticket. All others will be required to procure tickets.

Fees for the Spring Term—exclusive of Hospital and Demonstrator's Tickets—$20.

Certificates of attendance upon the Full Course will be considered the equivalent of time passed under the instruction of a medical practitioner during the Spring and Summer months of the year.

For the Annual Catalogue of the College, and for additional information concerning the Spring Session,

Address

Dr. E. POWELL, *45 S. Clark St.*

Students arriving in the City are requested to call at once upon the Janitor, Mr. CHARLES KEIL, at the College Building, corner of North Dearborn and Indiana Streets, Chicago.

